# Effects of dexmedetomidine as an adjuvant to ropivacaine or ropivacaine alone on duration of postoperative analgesia: A systematic review and meta-analysis of randomized controlled trials

**DOI:** 10.1371/journal.pone.0287296

**Published:** 2023-10-11

**Authors:** Fangzhou Li, Liang Guo, Zijin Huang, Fei Lin, Linghui Pan

**Affiliations:** 1 Department of Anesthesiology, Guangxi Medical University Cancer Hospital, Nanning, Guangxi Zhuang Autonomous Region, China; 2 Guangxi Key Laboratory for Basic Science and Prevention of Perioperative Organ Dysfunction, Nanning, Guangxi Zhuang Autonomous Region, China; 3 Guangxi Engineering Research Center for Tissue & Organ Injury and Repair Medicine, Nanning, Guangxi Zhuang Autonomous Region, China; 4 Guangxi Clinical Research Center for Anesthesiology, Nanning, Guangxi Zhuang Autonomous Region, China; Maulana Azad Medical College, INDIA

## Abstract

**Background:**

Ropivacaine is a long-acting local anesthetic that is used to treat postoperative pain. Adjuvant use of dexmedetomidine in regional anesthesia may prolong the duration of analgesia. The objective of this systematic review and meta-analysis was to investigate the duration and effect of ropivacaine alone vs. ropivacaine in combination with dexmedetomidine for postoperative analgesia.

**Methods:**

The PubMed, EMBASE, Web of Science, and Google Scholar databases were searched for randomized controlled trials (RCTs) of ropivacaine alone or ropivacaine in combination with dexmedetomidine for regional anesthesia. The primary outcome was duration of analgesia, defined as the time from onset of the block to the time of the first analgesic request or initial pain report. Secondary outcomes were duration of sensory block, duration of motor block, consumption of sufentanil for analgesia, length of hospital stay, and incidence of postoperative nausea and vomiting.

**Results:**

Eighteen studies with 1148 patients were included. Overall quality of the RCTs, as assessed by the Jadad scale, was high. The meta-analysis demonstrated that ropivacaine combined with dexmedetomidine significantly prolonged the duration of postoperative analgesia from local anesthetics compared to ropivacaine alone (WMD: 4.14h; 95%CI: 3.29~5.0h; *P*<0.00001; I^2^ = 99%). There was evidence of high heterogeneity between studies. The duration of sensory and motor block was significantly increased, and consumption of sufentanil for analgesia and the incidence of postoperative nausea and vomiting were significantly reduced in patients who received ropivacaine combined with dexmedetomidine compared to ropivacaine alone. There was no significant difference in length of hospital stay.

**Conclusions:**

Compared to ropivacaine alone, ropivacaine combined with dexmedetomidine significantly prolonged the duration of postoperative analgesia and sensory and motor block, and reduced consumption of sufentanil for analgesia and the incidence of postoperative nausea and vomiting, across an array of surgeries.

## Introduction

Ropivacaine, one of the most common long-acting local anesthetics, is widely used to treat postoperative pain. The use of ropivacaine for regional anesthesia promotes patient recovery after surgery by facilitating earlier ambulation [1], improving sleep quality [2], reducing opioid consumption [3], and decreasing gastrointestinal adverse reactions [4]. However, ropivacaine alone has a short duration when used for nerve block, usually lasting 9–14 hours, and its role in postoperative analgesia is limited. Local anesthetic nerve block with ropivacaine is concentration-dependent, and ropivacaine has an improved sensory versus motor block profile at lower concentrations. There remains an unmet clinical need to find a reliable strategy for prolonging the analgesic effect of ropivacaine after surgery.

Dexmedetomidine is a highly selective α_2_ adrenergic receptor agonist. Adjuvant use of dexmedetomidine in regional anesthesia may prolong the duration of analgesia [5] by inhibiting the production of C and Aδ fiber action potentials, enhancing the inhibition of local anesthetics on sodium ion channels, and/or reducing the release of epinephrine, substance P and other neurotransmitters [6]. Several clinical studies suggest that dexmedetomidine is effective when used as an adjuvant to regional anesthesia to prolong peripheral nerve block [7–10]; however, concerns for side effects and potential toxicity persist.

Analgesic effects with two synergistically interacting anesthetics should occur at lower doses. The safety and efficacy of dexmedetomidine as an adjuvant to ropivacaine have been investigated in randomized controlled trials (RCTs). To the authors’ knowledge, the results from these studies have not been quantitatively synthesized. The objective of this systematic review and meta-analysis was to investigate the duration and effect of ropivacaine alone vs. ropivacaine in combination with dexmedetomidine for postoperative analgesia.

## Materials and methods

### Research protocol and registration

This systematic review and meta-analysis adhered to the recommendations of the Cochrane Collaboration and is reported per the Preferred Reporting Items for Systematic Reviews and Meta-Analysis (PRISMA) guidelines [11]. The protocol is registered with the International Prospective Register of Systematic Reviews (CRD42022316688). The review did not include any human or animal experiments conducted by the authors and therefore does not require ethical review.

### Information sources

Two reviewers (L.F.Z. and H.Z.J.) independently searched the PubMed, EMBASE, Web of Science, and Google Scholar databases from inception to April 2022, for full text articles reporting RCTs of ropivacaine alone or ropivacaine in combination with dexmedetomidine for regional anesthesia. Searches were restricted to full text articles published in the English language. References of included studies were searched to identify additional relevant articles.

### Search strategy

Search terms included subject headings and free words. The search strategy for PubMed was: (((("Ropivacaine") AND ("Dexmedetomidine")) AND ("Regional anaesthesia" OR "Conduction Anesthesia" OR "Regional Anesthesia" OR "nerve block" OR "Nerve Blocks" OR "Nerve Blockade" OR "Nerve Blockades" OR "peripheral block" OR "Local Anesthesia" OR "Infiltration Anesthesia" OR "local infiltration anesthesia")) AND ("Post surgical Pain" OR "Post-operative Pain" OR "Postoperative Pain" OR "Chronic Postoperative Pain" OR "Persistent Postsurgical Pain" OR "Acute Postoperative Pain" OR "Perioperative analgesia" OR "Postoperative analgesia")) AND ("Randomized controlled trial" OR "Placebo" OR "Randomly" OR "Randomized").

### Eligibility criteria

Inclusion criteria were: 1) Population: adult patients aged >18 years receiving regional anesthesia with ropivacaine; 2) Intervention: addition of dexmedetomidine to ropivacaine for perioperative analgesia; 3) Comparator: ropivacaine alone for perioperative analgesia; and 4) Outcomes: (primary outcome) duration of postoperative analgesia, defined as the time from onset of the block to the time of the first analgesic request or initial pain report; (secondary outcomes) duration of sensory block, defined as the time from onset of the sensory nerve block to complete recovery of normal sensation (patient reported feelings of cold, heat and/or pain), duration of motor block, defined as the time from onset of the motor nerve block to complete recovery of normal motor function [12], length of hospital stay, consumption of sufentanil for analgesia, and incidence of postoperative nausea and vomiting. Exclusion criteria were 1) conference abstracts, reviews, and animal investigations; or 2) studies on intravenous regional anesthesia.

### Study selection

EndNote 9 was used to manage references. Two reviewers (L.F.Z. and H.Z.J.) independently examined titles and abstracts to select eligible studies and exclude duplicates. Full text articles, including supplementary materials, were retrieved and reviewed to determine which studies met the inclusion criteria. Discrepancies between reviewers were resolved by discussion and consensus, or through consultation with a third reviewer (L.F.).

### Data extraction

Two reviewers (L.F.Z. and G.L.) independently extracted data from the eligible studies, including study characteristics (publication year, subjects, research design, and outcomes), patients (demographic characteristics, sample size of each group, administration route, drug dose and concentration, and type of surgery), interventions, duration of analgesia, duration of sensory block, duration of motor block, length of hospital stay, analgesic consumption, and incidence of postoperative nausea and vomiting. Sample size, mean and standard deviation (SD) were recorded for continuous variables. Sample size and number of events (Yes/No) were recorded for dichotomous variables. Medians (quartile) and confidence intervals (CIs) were converted into means and SDs using methodology described in the Cochrane Handbook [13]. Study authors were contacted for missing data.

### Quality of evidence

Two reviewers (L.F.Z. and G.L.) independently assessed the methodological quality of each RCT using the Cochrane Collaboration’s Risk of Bias Tool [14] and Modified Jadad Scale (range, 0–7) [15]. Discrepancies between reviewers were resolved by discussion and consensus, or through consultation with a third reviewer (L.F.).

### Statistical analysis

Statistical analysis was performed using Review Manager 5.3 (Oxford, UK) and Stata 14.0. The effect measure for continuous outcomes was weighted mean difference (WMD) (SD with 95% CI). The effect measure for dichotomous outcomes was risk ratio (RR) (with 95% CI). Heterogeneity between studies was identified according to the inconsistency index and the Cochrane Q statistic (p<0.1 or *I*^*2*^≥50%). A fixed-effect model was used where there was evidence of low heterogeneity between studies (*P* > 0.1 or I^2^ < 50%), otherwise a random effects model was selected (*P* < 0.1 or I^2^ > 50%). Subgroup analysis and meta-regression were used to determine possible sources of heterogeneity. Sensitivity analysis, omitting one study at a time, was used to assess the stability of the results. A funnel plot, Egger’s regression intercept test, and the trim- and fill-method were used to evaluate publication bias.

## Results

### Study selection

**Fig 1:**
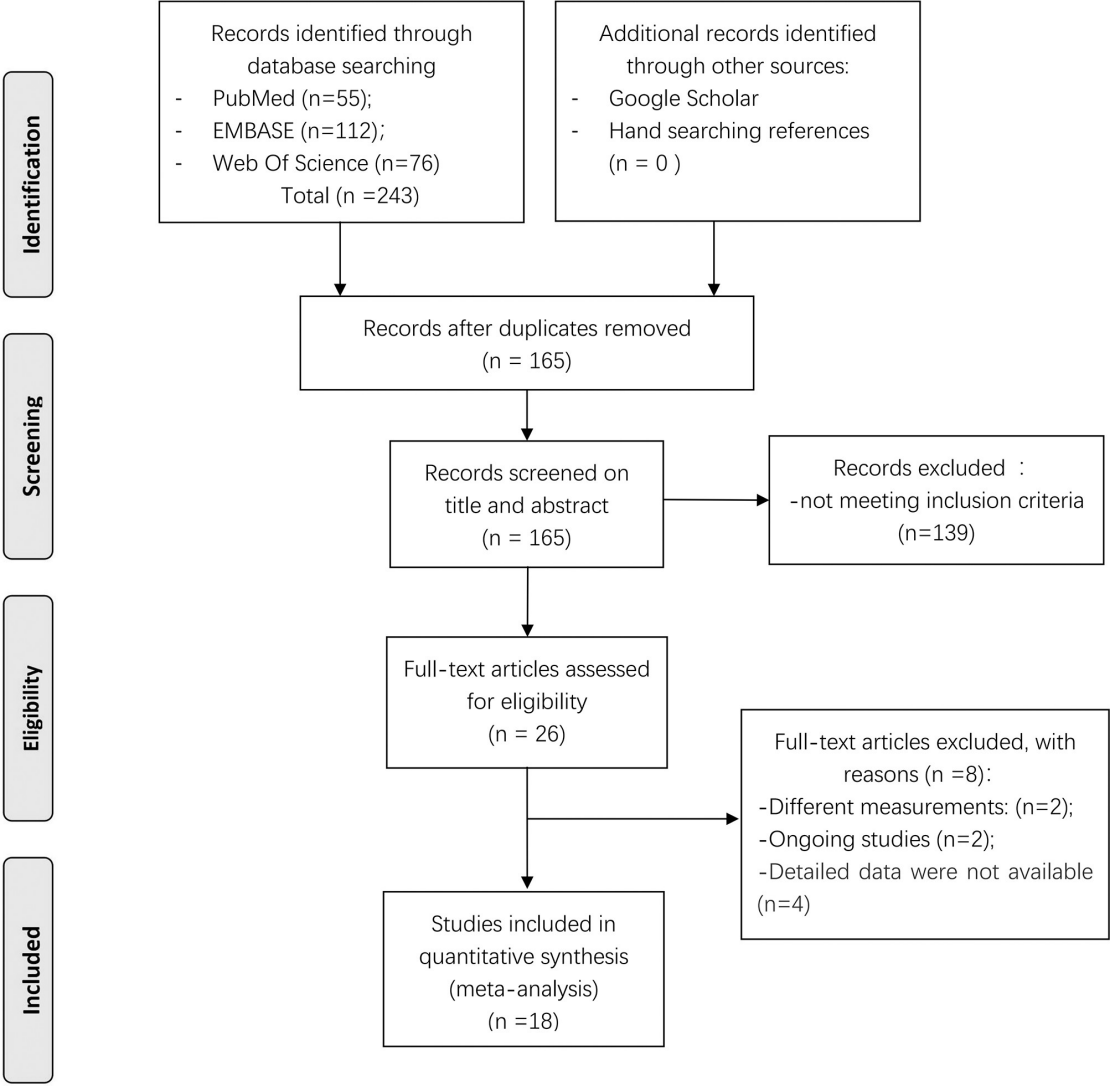
Flow chart for study selection.

The search identified 165 unique records. After screening titles and abstracts, 26 full-text articles were assessed for eligibility. Of these, eight articles were excluded, including two articles that did not report the outcome measures of interest, two articles that reported ongoing studies, and four articles that had missing data. Finally, 18 articles were included in the meta-analysis (**Fig 1**).

### Basic characteristics of included studies

Characteristics of the included studies are shown in **Table 1**. The studies were RCTs published between 2012 and 2021. The RCTs included a total of 1,148 patients, with a mean age of 45 years and American Society of Anesthesiologists (ASA) Physical Status Classification Grade I-III. 572 patients received ropivacaine alone, and 575 patients received ropivacaine in combination with dexmedetomidine [6, 12, 16–31]. Concentrations of ropivacaine ranged from 0.25% to 0.75%. In terms of the type of surgery, three RCTs [16–18] were conducted in patients undergoing thoracic surgery, ten RCTs [12, 20–28] were conducted in patients undergoing orthopedic surgery, two RCTs [6, 19] were conducted in patients undergoing breast surgery, and three RCTs [29–31] were conducted in patients undergoing abdominal surgery. Four RCTs [12, 21, 24, 28] used brachial plexus nerve block, four RCTs [16, 17, 22, 30] used paravertebral block, four RCTs [20, 23, 25–27] used lower limb nerve block, three RCTs [6, 18, 19] used intercostal nerve block, two RCTs [29, 31] used transversus abdominis plane (TAP) block and one RCT [27] used epidural block anesthesia.

**Table 1:** Characteristics of the included studies.

Study	Country	Number of patients Ropivacaine+DEX/Ropivacaine	Surgery	Intervention	Administration route	Other anaesthesia	Outcome
Sinha (2012)	India	29/29	Unilateral renal surgery	18 ml of ropivacaine 0.25% + 1μg/kg DEX	Paravertebral block	General anaesthesia	Duration of analgesia
Kaur (2014)	India	50/50	Lower limb orthopedic surgeries	150 mg of 0.75% ropivacaine + DEX (1 μg/kg)	Epidural anesthesia	None	Duration of analgesia; duration of sensory block; duration of motor block
Bangera (2016)	India	40/40	Upper limb surgeries	39 ml of 0.375% ropivacaine and DEX 1 μg/kg	Brachial plexus block	None	Duration of analgesia; duration of sensory block; duration of motor block
Panigrahi (2016)	India	20/20	Knee arthroscopy	20 ml of 0.2% ropivacaine + DEX 1μg/kg	Injected intraarticularly by the surgeon	Spinal anesthesia	Duration of analgesia
Sharma (2016)	India	25/25	Total knee replacement (TKR)	22 ml of 0.2% ropivacaine + 1.5 μg/kg DEX	Femoral nerve block	Spinal anesthesia	Duration of analgesia
Liu (2018)	China	57/57	Elective upper limb surgery	20 mL 0.375% ropivacaine +100ug DEX	Brachial plexus block	Phenobarbital sodium (0.1 g)	Duration of analgesia; duration of sensory block; duration of motor block; postoperative nausea
Jung (2018)	South Korea	25/23	Arthroscopic shoulder surgery	20 mL of 0.5% ropivacaine+2 mL of DEX 1 ug/kg	Brachial plexus block	General anaesthesia	Duration of analgesia; duration of sensory block; duration of motor block
Kaur (2017)	India	30/30	Oncological breast surgeries	30 ml of ropivacaine 0.25% + DEX 1 μg/kg	Pectoral nerve block (Pecs)	General anaesthesia	Duration of analgesia; postoperative nausea
Kundra (2019)	India	30/30	Hemi-arthroplasty	3 mg/kg of ropivacaine+1 μg/kg DEX	Fascia iliaca compartment block (FICB)	Spinal anesthesia	Duration of analgesia
Li (2019)	China	29/28	Elective posterior lumbar interbody fusion (PLIF) surgery for symptomatic single-level (L4-L5 or L5-S1) stenosis	20 mL 0.5% ropivacaine +1 ug/kg of DEX	Injected the skin and subcutaneous tissues	General anaesthesia	Duration of analgesia
Sharma (2019)	Nepal	30/30	Upper limb surgeries	30 ml of 0.5% Ropivacaine + 0.75 ug/kg DEX	Brachial plexus block	None	Duration of analgesia; duration of sensory block
Zhang (2019)	China	20/20	Thoracoscopic pneumonectomy	28 mL of ropivacaine 0.5%+DEX 1 μg/kg in 2 mL.	Intercostal nerve block (INB)	General anaesthesia	Duration of analgesia; postoperative nausea
Xu (2018)	China	30/30	Emergency laparotomy (gastric or intestinal perforation, intestinal obstruction or enterectomy)	40 mL of 0.25% ropivacaine+0.5μg/kg DEX	Transversus abdominis plane (TAP) and rectus sheath (RS) blocks	General anaesthesia	Duration of analgesia; duration of sensory block; length of hospital stay; analgesic consumption (sufentanil)
Yao (2020)	China	50/50	Lumpectomy	DEX 0.5μg/kg + intercostal nerve block with 0.5% ropivacaine	INB	Epidural block	Duration of analgesia
Pan (2020)	China	30/30	Elective laparoscopic colectomy	20 ml of 0.375% ropivacaine + 2 ml DEX (0.5 μg/kg)	Transversus abdominis plane (TAP)	General anaesthesia	Duration of analgesia; duration of sensory block; postoperative nausea and vomiting; length of hospital stay
Wang (2021)	China	30/30	Open thoracotomy of thoracic esophageal cancer	28 mL of 0.5% ropivacaine + 0.5 μg/kg in 2 mL of DEX	Erector spinae plane block (ESPB)	General anaesthesia	Duration of analgesia; postoperative nausea and vomiting; analgesic consumption(sufentanil)
Zha (2021)	China	20/20	VATS	10ml 0.5% ropivacaine+1ug/kg DEX	Thoracic paravertebral block (TPVB)	General anaesthesia	Duration of analgesia; postoperative nausea and vomiting; analgesic consumption(sufentanil)
Jin (2021)	China	30/30	Total knee arthroplasty (TKA)	0.25% ropivacaine 40 mL+ 0.5 ug/kg DEX	Femoral nerve block and sciatic nerve block	General anaesthesia	Duration of analgesia; length of hospital stay

### Risk of bias assessment

Most RCTs were categorized as low risk of bias (**S1 and S2 Figs, S2 Table**). Eight RCTs did not describe allocation concealment. Four RCTs did not describe methods of blinding patients and personnel. Eight RCTs did not describe blinding of the outcome assessment.

### Duration of analgesia

#### Pooled effect size

**Fig 2:**
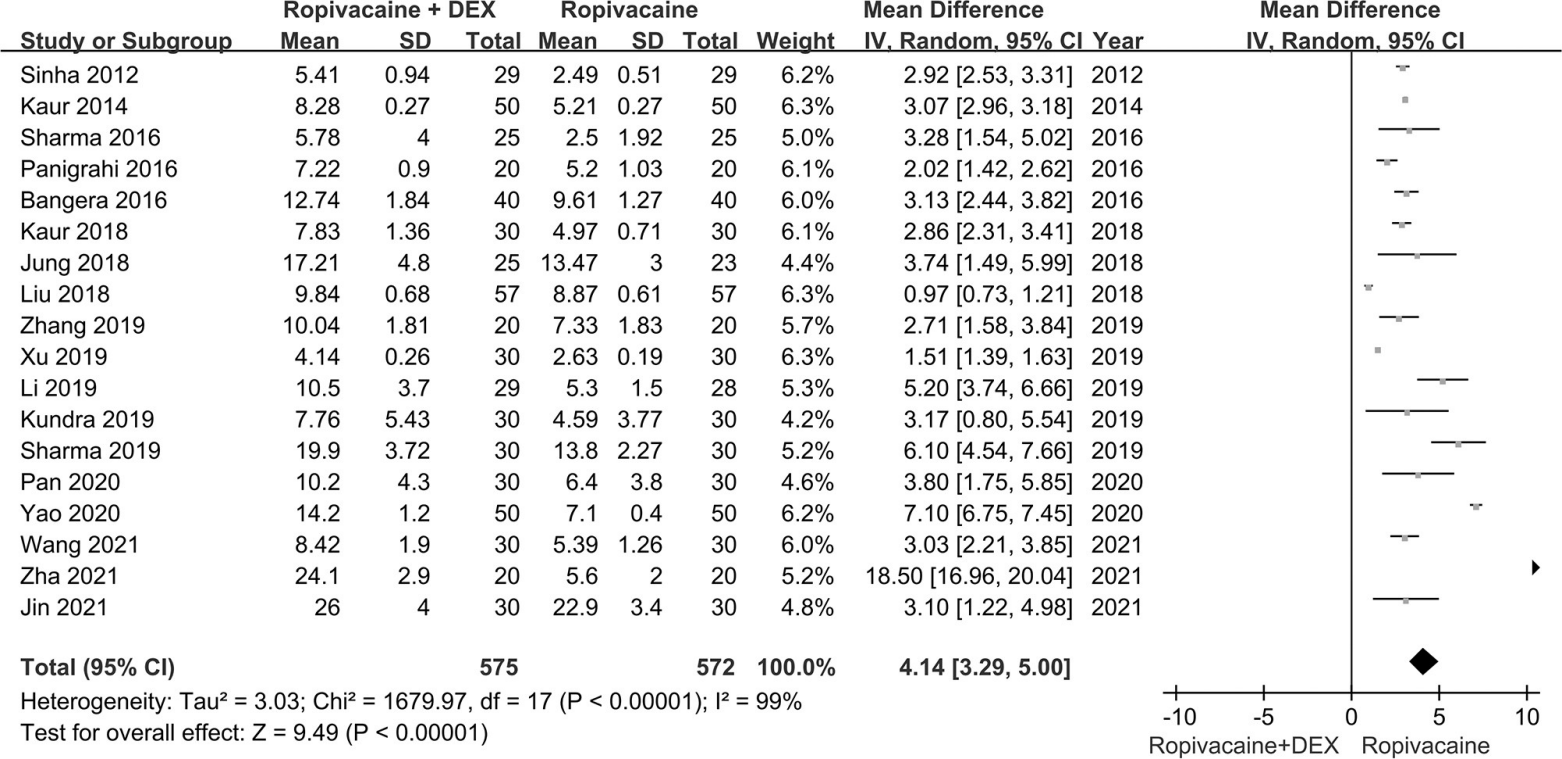
Forest plot for duration of postoperative analgesia (h), hour.

Duration of postoperative analgesia was reported in all 18 RCTs. The meta-analysis demonstrated that ropivacaine combined with dexmedetomidine significantly prolonged the duration of postoperative analgesia from local anesthetics compared to ropivacaine alone (WMD: 4.14h; 95%CI: 3.29~5.0h; *P<*0.00001; I^2^ = 99%) (**Fig 2**).

#### Heterogeneity

There was evidence of high heterogeneity between studies (**Fig 2**), Sensitivity analysis implied the results were relatively stable, and heterogeneity was not due to any individual study (**S3 Fig**). Meta-regression analysis of route of administration, drug concentration, and type of surgery implied the source of heterogeneity could not be explained by these three variables (**S4 Fig**).

#### Publication bias

The funnel plot and Begg’s funnel plot are shown in **S5 and S6 Figs**. The trim and fill method showed publication bias had no significant effect on the outcomes of this review (**S7 Fig**).

#### Subgroup analysis

**Fig 3:**
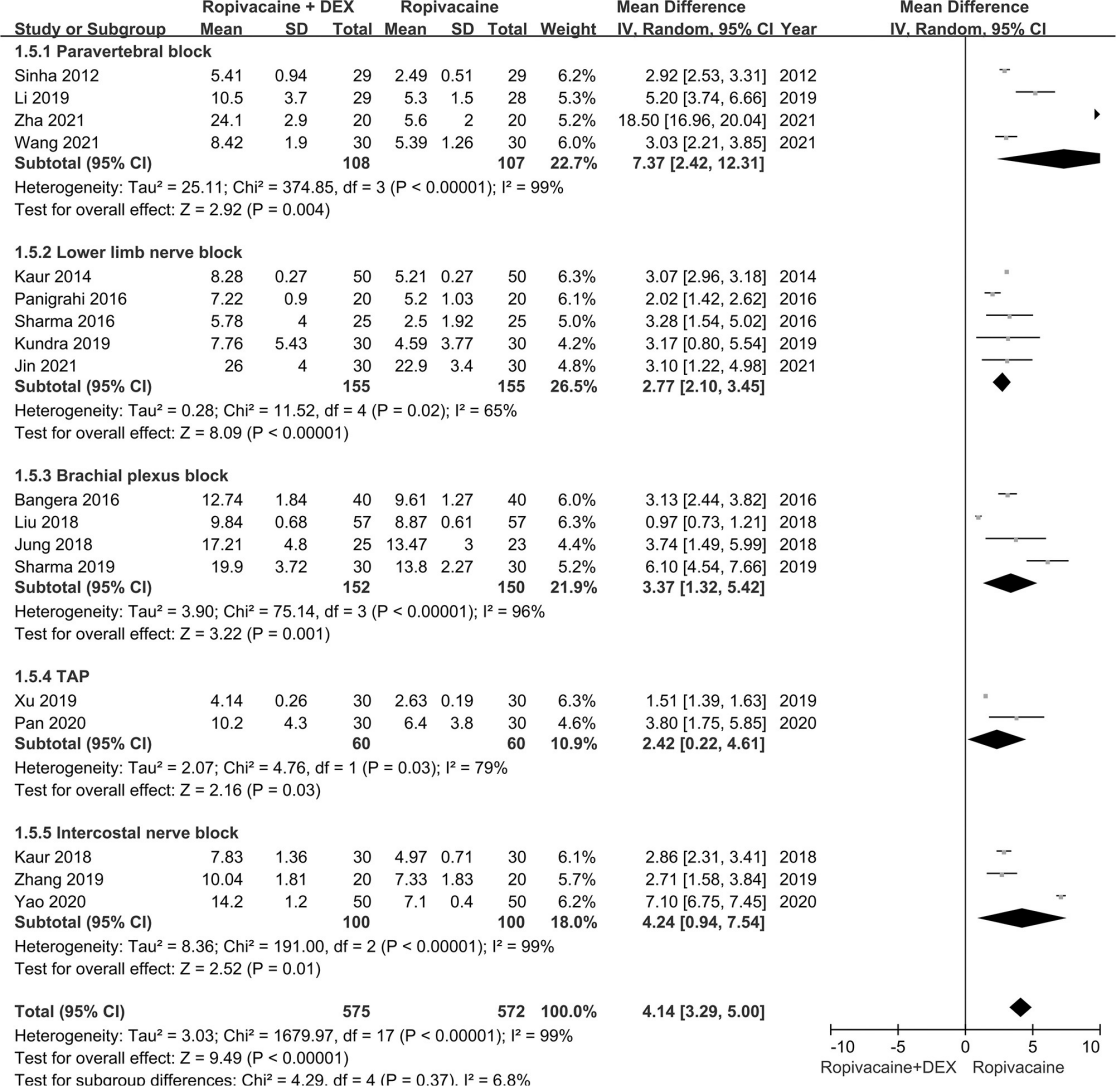
Subgroup analysis stratified by route of administration.

Subgroup analysis was stratified by route of administration. The difference in mean duration of postoperative analgesia between patients who received ropivacaine in combination with dexmedetomidine or ropivacaine alone was 7.37h (95%CI: 2.42–12.31h; *P* = 0.004) for paravertebral block, 2.77h (95%CI: 2.10–3.45h; *P<*0.0001) for lower limb nerve block, 3.37h (95%CI: 1.32–5.42h; *P* = 0.001) for brachial plexus nerve block, 2.42h (95%CI: 0.22–4.61h; *P* = 0.03) for TAP block, and 4.24h (95%CI: 0.94–7.54h; *P* = 0.01) for intercostal nerve block. The difference in mean duration of postoperative analgesia between patients who received ropivacaine in combination with dexmedetomidine or ropivacaine alone was similar across subgroups (*P* = 0.37) (**Fig 3**) and was not influenced by type of surgery (*P* = 0.07) **(S8 Fig)** or use of general anesthesia (*P* = 0.32) **(S9 Fig)**.

### Duration of sensory and motor block

**Fig 4:**
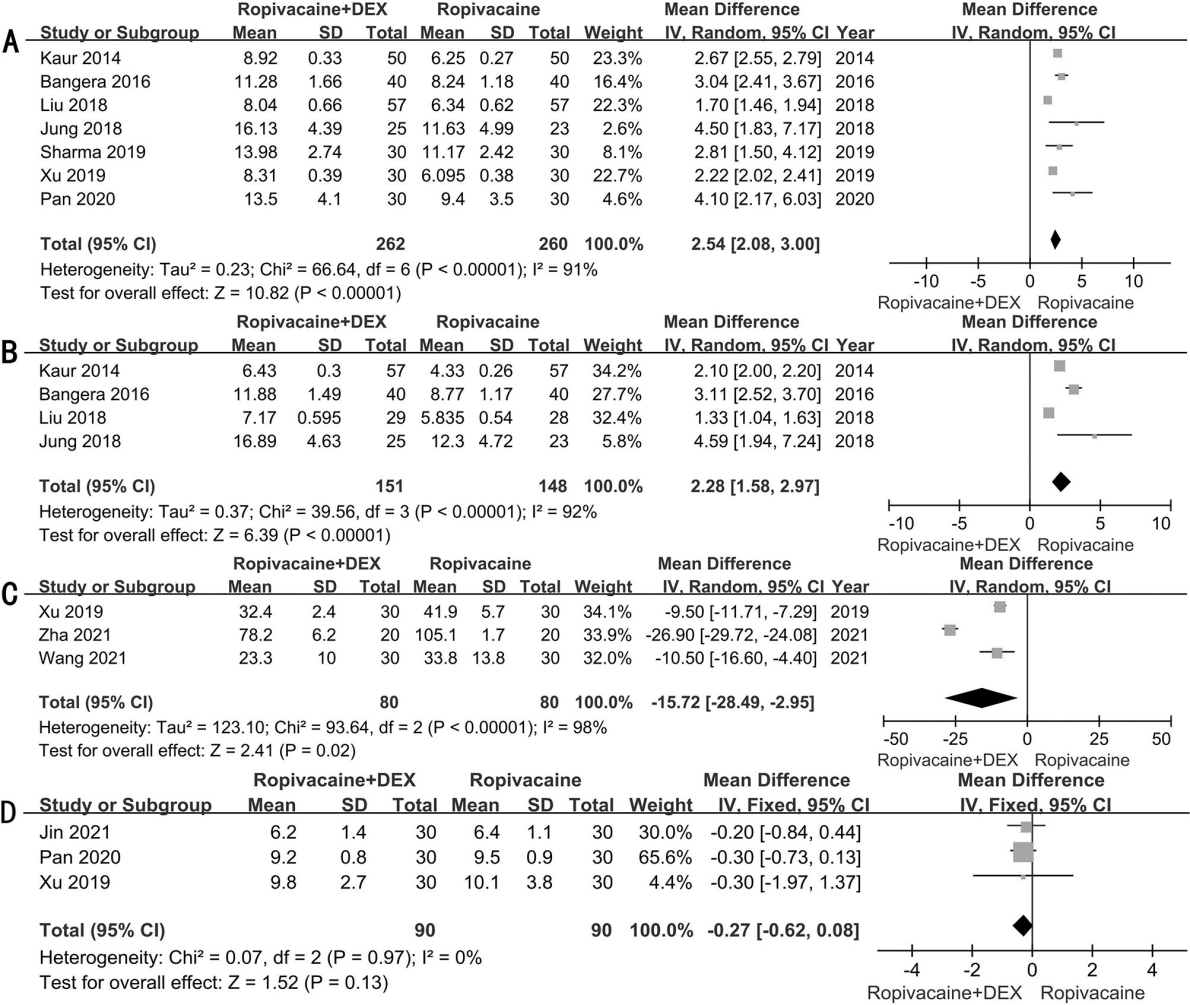
Forest plots for duration of sensory block(A); motor block (B); analgesic consumption of sufentanil (C) and length of hospital stay (D).

Duration of sensory block was reported in seven RCTs [12, 21, 24, 27–29, 31]. The meta-analysis demonstrated that ropivacaine combined with dexmedetomidine significantly prolonged the duration of sensory block compared to ropivacaine alone (WMD: 2.54h; 95%CI: 2.08–3.0h; *P*<0.0001) (**Fig 4A**). Duration of motor block was reported in four RCTs [12, 24, 27, 28]. The meta-analysis demonstrated that ropivacaine combined with dexmedetomidine significantly prolonged the duration of motor block compared to ropivacaine alone (WMD: 2.28h; 95%CI: 1.58–2.97h; *P*<0.0001) (**Fig 4B**).

### Analgesic consumption of sufentanil

Consumption of sufentanil for analgesia was reported in three RCTs [16, 17, 29]. The meta-analysis demonstrated a significant reduction in the consumption of sufentanil in patients who received ropivacaine combined with dexmedetomidine compared to ropivacaine alone (WMD:-15.72ug; 95%CI:-28.49~-2.95ug; *P* = 0.02). **(Fig 4C).**

### Length of hospital stay

Length of hospital stay was reported in three RCTs [10, 29, 31]. The meta-analysis demonstrated no significant difference in length of hospital stay for patients who received ropivacaine combined with dexmedetomidine compared to ropivacaine alone (WMD: -0.27d; 95%CI: -0.62–0.08d; *P* = 0.13) (**Fig 4D**).

### Postoperative nausea and vomiting

**Fig 5:**
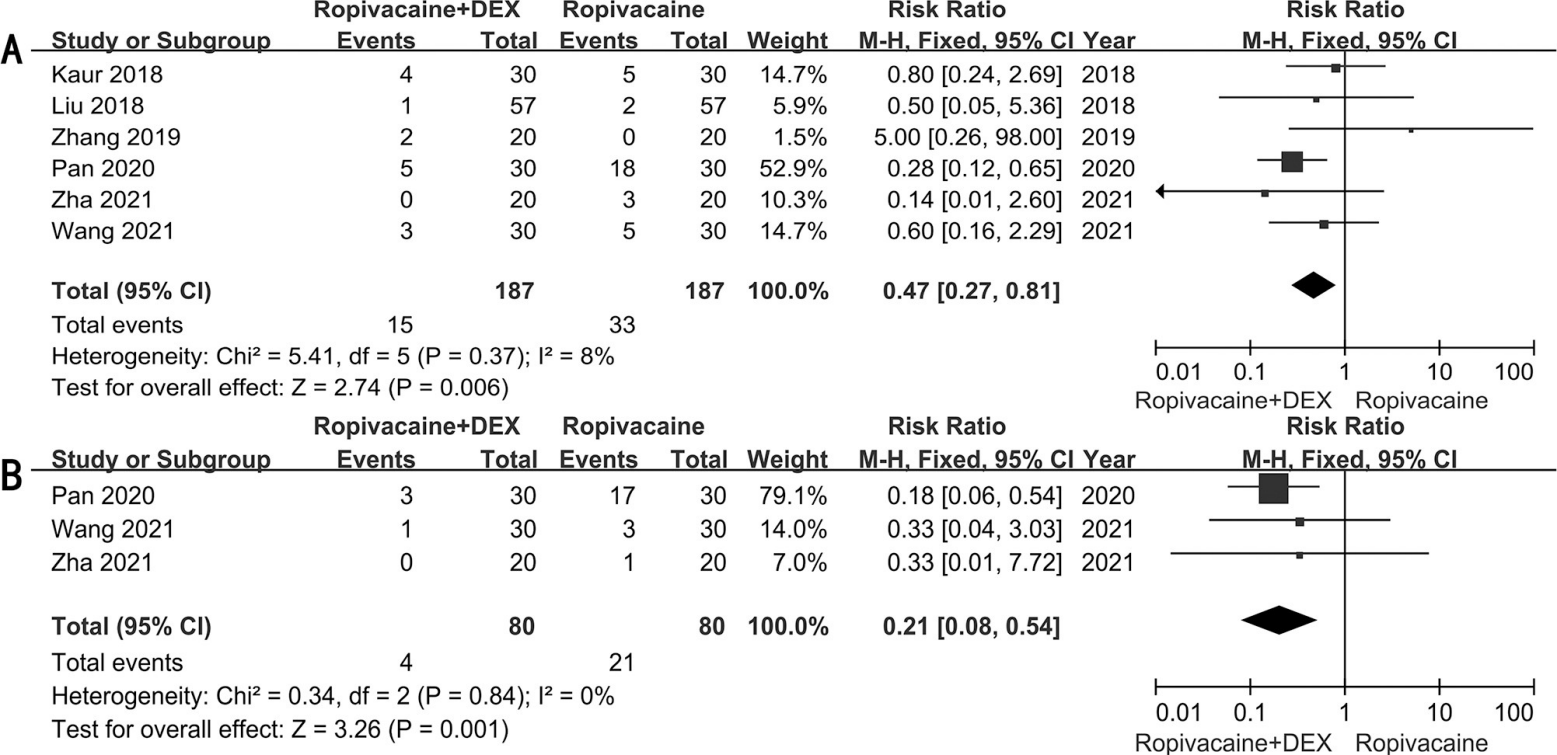
Forest plots for postoperative nausea (A) and vomiting (B).

The incidence of postoperative nausea was reported in six RCTs [16–19, 24, 31], and the incidence of postoperative vomiting was reported in three RCTs [16, 17, 31]. The meta-analysis demonstrated a significant reduction in the incidence of postoperative nausea and vomiting in patients who received ropivacaine combined with dexmedetomidine compared to ropivacaine alone (nausea: RR: 0.47; 95% CI: 0.27 ~ 0.81; *P* = 0.006; vomiting: RR: 0.21; 95% CI: 0.08 ~ 0.54; *P* = 0.001) (**Fig 5**).

## Discussion

This meta-analysis of 18 RCTs, which included a total of 1,148 patients, investigated the duration and effect of ropivacaine alone vs. ropivacaine in combination with dexmedetomidine for postoperative analgesia. Findings showed that ropivacaine combined with dexmedetomidine significantly prolonged the duration of postoperative analgesia and sensory and motor block, and reduced consumption of sufentanil for analgesia and the incidence of postoperative nausea and vomiting compared to ropivacaine alone.

Ropivacaine combined with dexmedetomidine significantly prolonged the duration of postoperative analgesia compared to ropivacaine alone irrespective of route of administration, type of surgery, degree of postoperative pain, and concentration and dose of anesthetic drugs. Among the RCTs included in this review, brachial plexus block was most often employed for upper limb surgery, intercostal nerve block and paravertebral block were most often employed for thoracic surgery, and TAP block was most often employed for abdominal surgery. Combination treatment most commonly consisted of 0.5% ropivacaine +0.5–1μg/kg DEX. Lower limb surgery was usually joint replacement in elderly patients, which was associated with surgical trauma and obvious postoperative pain. 0.5% ropivacaine +0.5–1μg/kg DEX prolonged postoperative analgesia in these patients, and the low concentration of ropivacaine did not interfere with early ambulation. In patients undergoing chest and limb surgery, dexmedetomidine as an adjuvant to ropivacaine prolonged the duration of postoperative analgesia for 3–6 hours compared to ropivacaine alone. For abdominal surgery, 0.25%-0.375% ropivacaine combined with 0.5μg/kg DEX only prolonged postoperative analgesia by 2–3 hours compared to ropivacaine alone, likely because of the severity of visceral pain associated with this procedure. Our data imply that dexmedetomidine as an adjuvant to ropivacaine can optimize postoperative pain management in accordance with the “minimal use” or “opioid sparing” management concept proposed by the enhanced recovery after surgery (ERAS) protocol [32, 33].

Our pooled analyses showed that ropivacaine combined with dexmedetomidine significantly prolonged the duration of postoperative analgesia compared to ropivacaine alone; however, findings across individual studies varied. In chest surgery, these disparate results may be explained by differences in sample size, patient age and the use of the long-acting opioid analgesic sufentanil for patient-controlled analgesia (PCA). In upper limb surgery, these disparate results may be explained by differences in the volume and concentration of ropivacaine and dexmedetomidine and the use of nerve stimulation or ultrasound guided block.

Poor management of postoperative pain has been associated with increased morbidity, including impaired function and quality of life, prolonged opioid use, delayed recovery, and high health-care costs [34]. In particular, postoperative pain can promote the release of catecholamine from sympathetic nerve endings and the adrenal medulla, which activates the renin-angiotensin system. Catecholamine increases heart rate, myocardial oxygen consumption, and peripheral resistance, while angiotensin II can cause systemic vasoconstriction, resulting in increased blood pressure, tachycardia, arrhythmias, and myocardial ischemia in some patients [35, 36].

Dexmedetomidine as an adjuvant to ropivacaine for postoperative pain management has the potential to reduce this morbidity [5]. Other methods for reducing postoperative pain in clinical practice include oral or intravenous painkillers, PCA, music therapy, and acupressure. Therapeutic efficacy of these methods in reducing postoperative pain requires evaluation in RCTs.

This systematic review and meta-analysis adhered to the recommendations of the Cochrane Collaboration and is reported per the PRISMA guidelines; however, it has some limitations. First, there was evidence of high heterogeneity between studies, but the source could not be identified. Second, there was potential publication bias, which may be partly attributed to the tendency to publish positive results; and third, searches were restricted to full text articles published in the English language. Although there were no restrictions on region during the search, all included studies originated in China and other Asian countries (Nepal, India, and South Korea).

## Conclusion

Ropivacaine combined with dexmedetomidine significantly prolonged the duration of postoperative analgesia and sensory and motor block, and reduced consumption of sufentanil for analgesia and the incidence of postoperative nausea and vomiting, compared to ropivacaine alone, across an array of surgeries.

## Supporting information

S1 Table PRISMA checklist. (DOCX)

S2 Table Modified Jadad score. (DOCX)

S3 Table Search strategy. Detailed search strategy for PubMed, Embase and Web of Science.
(DOCX)

S4 Table Results of data collection and summary. Data collection results of meta-analysis based on included articles.
(XLSX)

S1 Fig Evaluation of risk of bias. (DOCX)

S2 Fig Risk of bias. (DOCX)

S3 Fig Sensitivity analysis for duration of postoperative analgesia. (DOCX)

S4 Fig Meta-regression analysis. (DOCX)

S5 Fig Begg’s funnel plot of publication bias. (PDF)

S6 Fig Funnel plot of publication bias. (DOCX)

S7 Fig Trim- and fill-method for publication bias. (DOCX)

S8 Fig Subgroup analysis stratified by type of surgery. (PDF)

S9 Fig Subgroup analysis stratified by presence/absence of general anesthesia. (PDF)
