# Supplementary material for: Effects of dexmedetomidine as an adjuvant to ropivacaine or ropivacaine alone on duration of postoperative analgesia: A systematic review and meta-analysis of randomized controlled trials
Source: PLoS One. 2023 Oct 11;18(10):e0287296. doi: 10.1371/journal.pone.0287296 (PMC10566714; doi:10.1371/journal.pone.0287296)
Supplement: S2 Table — (DOCX) [file pone.0287296.s002.docx]

**Supporting information 4. The quality assessment of RCTs—Modified Jadad Score**

| Author name (Year) | Randomization ^a^ | Concealment of allocation ^b^ | Double blinding ^c^ | Withdrawals and dropouts ^d^ | Final score | Overall |
| --- | --- | --- | --- | --- | --- | --- |
| Sinha (2012) | 2 | 1 | 1 | 1 | 5 | high quality |
| Kaur (2014) | 2 | 1 | 1 | 1 | 5 | high quality |
| Bangera (2016) | 2 | 2 | 2 | 1 | 7 | high quality |
| Panigrahi (2016) | 2 | 2 | 2 | 1 | 7 | high quality |
| Sharma (2016) | 2 | 1 | 2 | 1 | 6 | high quality |
| Liu (2018) | 2 | 2 | 2 | 1 | 7 | high quality |
| Jung (2018) | 2 | 1 | 2 | 1 | 6 | high quality |
| Kaur (2018) | 2 | 1 | 1 | 1 | 5 | high quality |
| Kundra (2019) | 2 | 2 | 2 | 1 | 7 | high quality |
| Li (2019) | 2 | 1 | 2 | 1 | 6 | high quality |
| Sharma (2019) | 2 | 2 | 2 | 1 | 7 | high quality |
| Zhang (2019) | 2 | 2 | 2 | 1 | 7 | high quality |
| Xu (2019) | 2 | 2 | 2 | 1 | 7 | high quality |
| Yao (2020) | 2 | 1 | 1 | 1 | 5 | high quality |
| Pan (2020) | 2 | 1 | 2 | 1 | 6 | high quality |
| Wang (2021) | 2 | 2 | 2 | 1 | 7 | high quality |
| Zha (2021) | 2 | 2 | 2 | 1 | 7 | high quality |
| Jin (2021) | 2 | 2 | 2 | 1 | 7 | high quality |

Comments: ^a/b/c^: Appropriate --2 points; Not described --1 point; Inappropriate or unused --0 point; ^d^: Described—1 point; Not described --0 point; A final score of 1-3 is considered low quality and a score of 4-7 is considered high quality.
