# Supplementary material for: Effects of dexmedetomidine as an adjuvant to ropivacaine or ropivacaine alone on duration of postoperative analgesia: A systematic review and meta-analysis of randomized controlled trials
Source: PLoS One. 2023 Oct 11;18(10):e0287296. doi: 10.1371/journal.pone.0287296 (PMC10566714; doi:10.1371/journal.pone.0287296)
Supplement: S3 Table — Detailed search strategy for PubMed, Embase and Web of Science. (DOCX) [file pone.0287296.s003.docx]

**Table 1**

The complete search strategy of PubMed

| Number | Search Terms |
| --- | --- |
| 1# | “Ropivacaine” [Mesh] |
| 2# | “Dexmedetomidine” [Mesh] |
| 3# | “Regional anaesthesia” OR “Conduction Anesthesia” OR “Regional Anesthesia” OR “nerve block” OR “Nerve Blocks” OR “Nerve Blockade” OR “Nerve Blockades” OR “peripheral block” OR “Local Anesthesia” OR “Infiltration Anesthesia” OR “local infiltration anesthesia” |
| 4# | "Post surgical Pain" OR "Post-operative Pain" OR "Postoperative Pain" OR "Chronic Postoperative Pain" OR "Persistent Postsurgical Pain" OR "Acute Postoperative Pain" OR "Perioperative analgesia" OR "Postoperative analgesia" |
| 5# | “Randomized controlled trial” OR “Placebo” OR “Randomly” OR “Randomized” |
| 6# | 1 AND 2 AND 3 AND 4 AND 5 |

**Table 2**

The complete search strategy of Embase

| Number | Search Terms |
| --- | --- |
| 1# | “Ropivacaine” |
| 2# | “Dexmedetomidine” |
| 3# | “Regional anaesthesia” OR “Conduction Anesthesia” OR “Regional Anesthesia” OR “nerve block” OR “Nerve Blocks” OR “Nerve Blockade” OR “Nerve Blockades” OR “peripheral block” OR “Local Anesthesia” OR “Infiltration Anesthesia” OR “local infiltration anesthesia” |
| 4# | “Post surgical Pain” OR “Post operative Pain” OR “Chronic Postoperative Pain” OR “Persistent Postsurgical Pain” OR “Acute Postoperative Pain” OR “Perioperative analgesia” OR “Postoperative analgesia” |
| 5# | “Randomized controlled trial” OR “Placebo” OR “Randomly” OR “Randomized” |
| 6# | 1 AND 2 AND 3 AND 4 AND 5 |

**Table 3**

The complete search strategy of Web of Science

| Number | Search Terms |
| --- | --- |
| 1# | TS=(“Ropivacaine”) |
| 2# | TS=(“Dexmedetomidine”) |
| 3# | TS= (“Regional anaesthesia” OR “Conduction Anesthesia” OR “Regional Anesthesia” OR “nerve block” OR “Nerve Blocks” OR “Nerve Blockade” OR “Nerve Blockades” OR “peripheral block” OR “Local Anesthesia” OR “Infiltration Anesthesia” OR “local infiltration anesthesia”) |
| 4# | TS= ("Post surgical Pain" OR "Post-operative Pain" OR "Postoperative Pain" OR "Chronic Postoperative Pain" OR "Persistent Postsurgical Pain" OR "Acute Postoperative Pain" OR "Perioperative analgesia" OR "Postoperative analgesia") |
| 5# | TS=(“Randomized controlled trial” OR “Placebo” OR “Randomly” OR “Randomized”) |
| 6# | 1 AND 2 AND 3 AND 4 AND 5 |
