# Supplementary material for: Effects of dexmedetomidine as an adjuvant to ropivacaine or ropivacaine alone on duration of postoperative analgesia: A systematic review and meta-analysis of randomized controlled trials
Source: PLoS One. 2023 Oct 11;18(10):e0287296. doi: 10.1371/journal.pone.0287296 (PMC10566714; doi:10.1371/journal.pone.0287296)
Supplement: S1 Fig — (DOCX) [file pone.0287296.s005.docx]

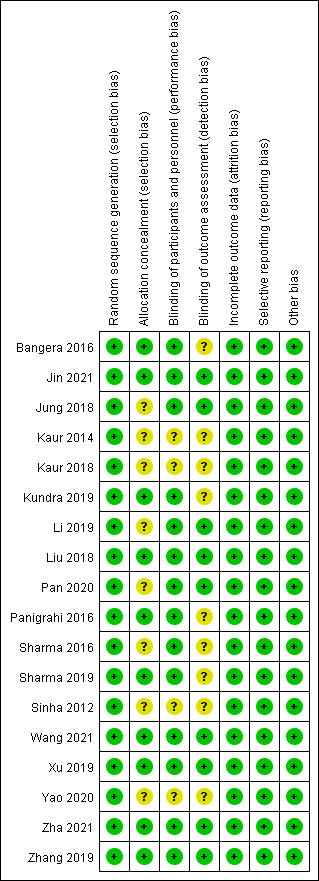


Supporting information 2: Evaluation of risk of bias for each included study. Green circles indicate low risk of bias, red circles indicate high risk of bias, yellow circles indicate unclear risk of bias.
