# Supplementary figures and images for: Effects of dexmedetomidine as an adjuvant to ropivacaine or ropivacaine alone on duration of postoperative analgesia: A systematic review and meta-analysis of randomized controlled trials
Source: PLoS One. 2023 Oct 11;18(10):e0287296. doi: 10.1371/journal.pone.0287296 (PMC10566714; doi:10.1371/journal.pone.0287296)

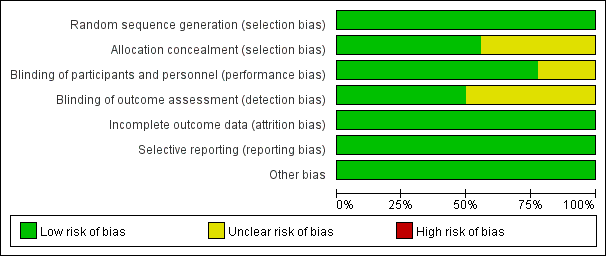


Supporting information 3: Risk of bias graph

Supplement: S2 Fig — (DOCX) [file pone.0287296.s006.docx]

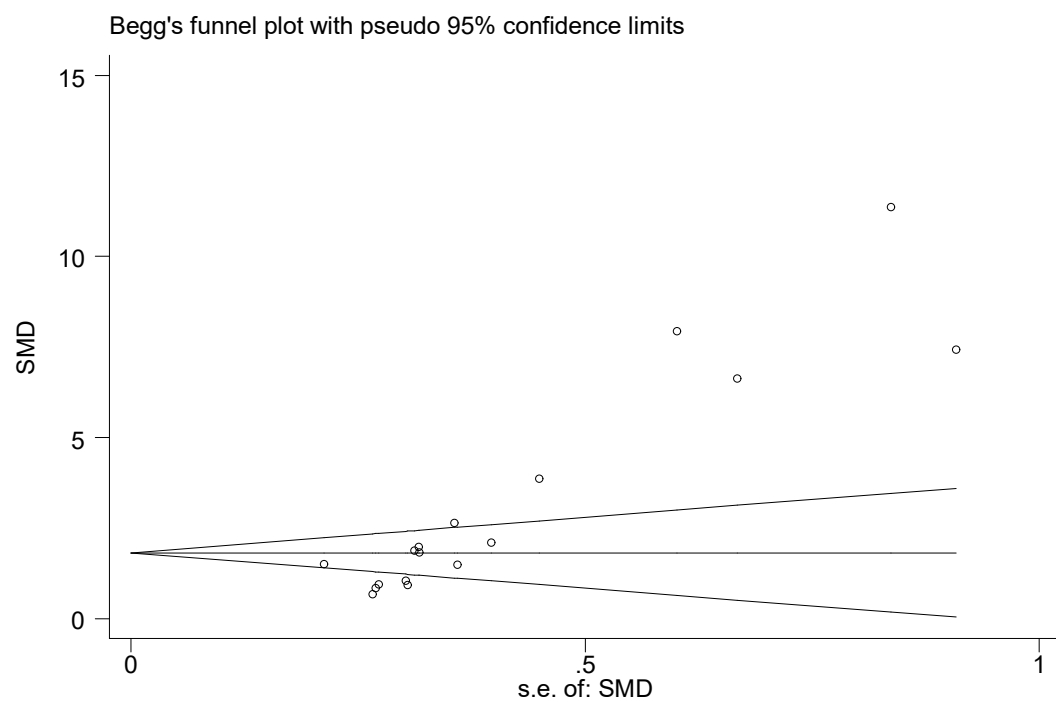

Supporting information 7 : Begg's funnel plot of publication bias.

Supplement: S5 Fig — (PDF) [file pone.0287296.s009.pdf]

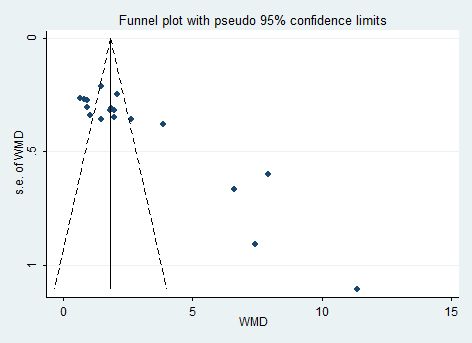


Supporting information 8: Funnel plot of publication bias

Supplement: S6 Fig — (DOCX) [file pone.0287296.s010.docx]

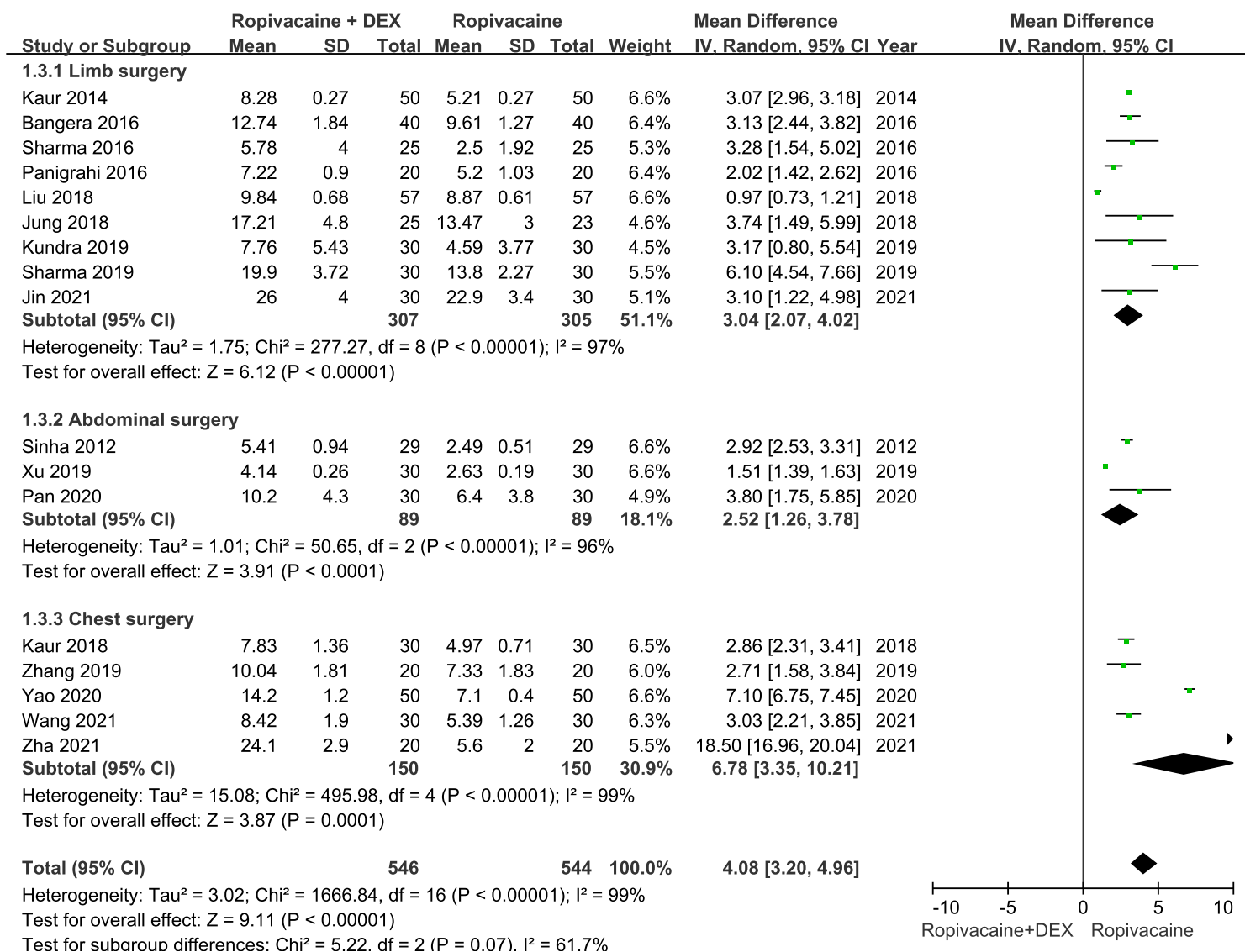

Supplement: S8 Fig — (PDF) [file pone.0287296.s012.pdf]

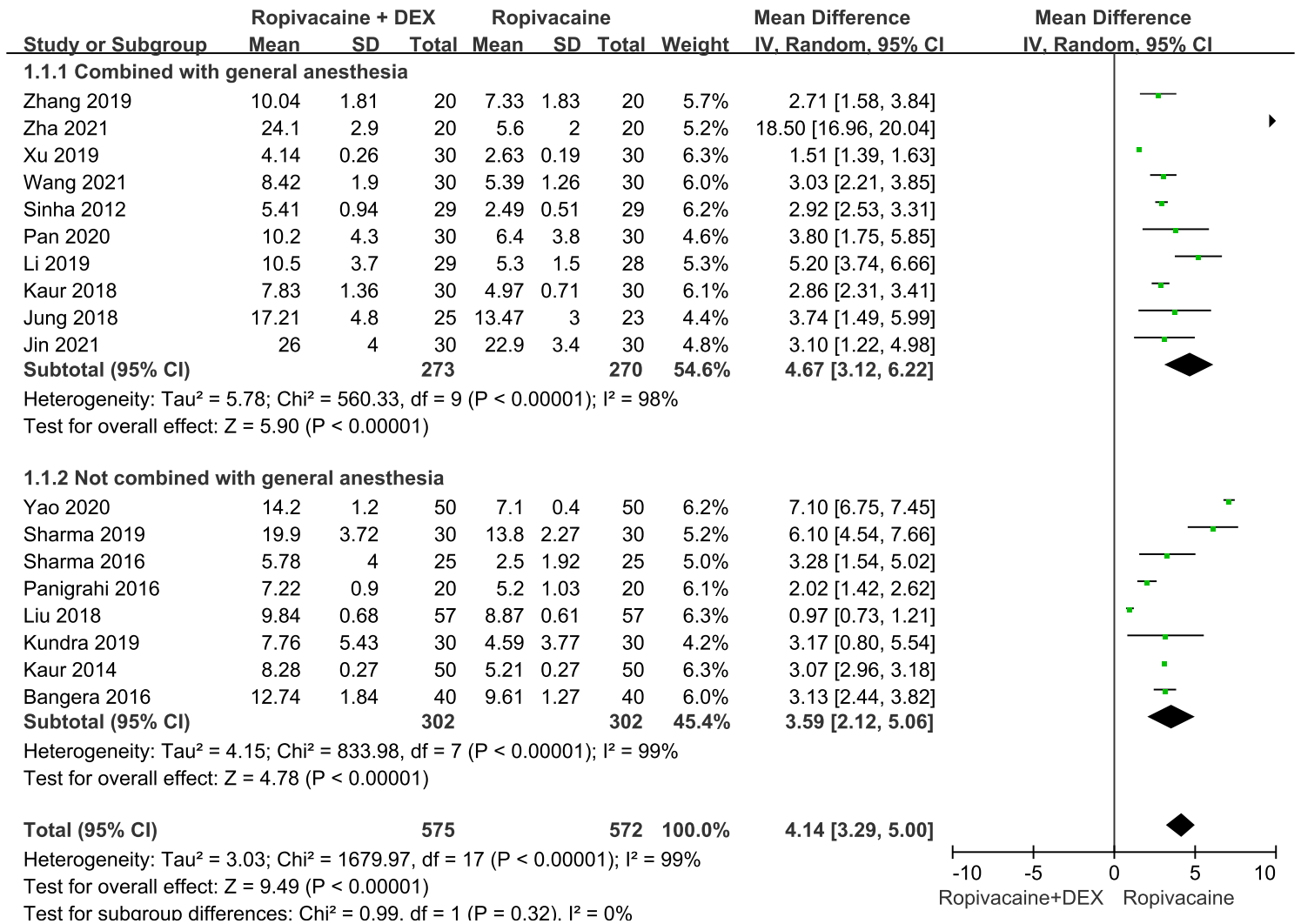

Supplement: S9 Fig — (PDF) [file pone.0287296.s013.pdf]
