# Supplementary material for: Effects of dexmedetomidine as an adjuvant to ropivacaine or ropivacaine alone on duration of postoperative analgesia: A systematic review and meta-analysis of randomized controlled trials
Source: PLoS One. 2023 Oct 11;18(10):e0287296. doi: 10.1371/journal.pone.0287296 (PMC10566714; doi:10.1371/journal.pone.0287296)
Supplement: S7 Fig — (DOCX) [file pone.0287296.s011.docx]

Supporting information 9: Trim- and fill-method for publication bias
